# *Bartonella* spp. Bacteremia and Rheumatic Symptoms in Patients from Lyme Disease–endemic Region

**DOI:** 10.3201/eid1811.120675

**Published:** 2012-11

**Authors:** C. Ben Beard, Christina A. Nelson, Paul S. Mead, Lyle R. Petersen

**Affiliations:** Author affiliation: Centers for Disease Control and Prevention, Fort Collins, CO, USA

**Keywords:** Bartonella, bacteremia, blood, arthritis, myalgia, PCR, DNA sequencing, bacteria, Lyme disease, rheumatic, rheumatologic

**To the Editor:** We believe the recent article by Maggi et al. (*1*) contains serious flaws in content and underlying message, including a poorly defined study population, lack of appropriate controls, improper use of the term bacteremia, and incongruent laboratory findings. Selection criteria were vague: the authors state only that participants were a “biased” collection of “patients selected by a rheumatologist,” with no control population included for comparison. The diagnosis of Lyme disease and other previously diagnosed conditions was solely by self-report. Although blood samples were collected from every participant, the authors apparently neglected to perform standardized testing for *Borrelia burgdorferi* or other conditions.

The term “bacteremia” signifies presence of viable bacteria in the bloodstream, which is not substantiated solely by a positive PCR result. True bacteremia was documented in only 1.7% of participants from whom a viable *Bartonella* species isolate was cultured, rather than the purported 41.1% of participants.

Surprisingly, many participants whose PCR results were positive for *Bartonella* spp. had no serologic evidence of infection (e.g., 82.5% of samples that had positive PCR results for *Bartonella henselae* were not seroreactive). Although anergy has been reported, samples from most immunocompetent and immunocompromised patients infected with *Bartonella* spp. are seroreactive (*2*–*4*), calling into question the authors’ findings. Furthermore, 24% of samples that were positive by PCR revealed no identifiable *Bartonella* spp. by DNA sequencing; these participants should have been excluded from analysis.

Maggi et al. hypothesize that *Bartonella* spp. infection is causally related to a variety of chronic ailments. In fact, there was no association within the study population between positive *Bartonella* spp. PCR results and chronic illness, self-reported Lyme disease, or even a prior diagnosis of bartonellosis.

Efforts to define the clinical and public health importance of *Bartonella* spp. require scientific rigor. The above issues challenge the validity of the study, and results should be interpreted with caution.
