# Supplementary material for: Bartonella spp. Bacteremia and Rheumatic Symptoms in Patients from Lyme Disease–endemic Region
Source: Emerg Infect Dis. 2012 Nov;18(11):1918–9. doi: 10.3201/eid1811.120675 (PMC3559143; doi:10.3201/eid1811.120675)
Supplement: Related texts

# Reply: *Bartonella* spp. Bacteremia and Rheumatic Symptoms in Patients from Lyme Disease–endemic Region

**Authors:** Didier Raoult

**Affiliations:** Author affiliation: Aix Marseille Université, Marseilles, France

**To the Editor:** Some chronic diseases, including multiple sclerosis, chronic arthritis (*1*), cognitive disorders, and chronic fatigue remain unexplained, yet patients and patient advocacy groups are anxious to find an explanation and a cure. For 50 years, zoonotic agents have been wrongly considered as the cause of many of these diseases because diagnoses were based on results of serologic tests with low specificity. In France, at the beginning of my career, serologic testing for rickettsia was used as a diagnostic tool for many of these diseases and prompted inappropriate antimicrobial drug use because micro-agglutination on a slide is a nonspecific serologic technique (*2*). I had a hard time reversing this practice.

Results of serologic testing for nanobacteria were also unconfirmed because they were based on nonspecific antibodies (*3*). Results of Lyme disease serologic tests lacking specificity were also associated with these chronic diseases and led to the same results and conflicts between the Infectious Diseases Society of America and alternative users of *Borrelia burgdorferi* diagnostic tests (*4*). Currently, Google search pages display more results for alternative interpretations than for scientific information. Again, I have tried to limit the damages in France without success (*5*).

Now the *Bartonella* spp. appear as the new candidates to explain chronic illness (*1*). Once more, I am confronted with the problem in France. Some patients whose test results are negative in my laboratory were tested at the College of Veterinary Medicine, North Carolina State University, Raleigh, NC, USA (*3*) and received positive results (using a technique that I have not been able to reproduce). Now one of my patients is arguing and menacing because I do not confirm his infection by *Bartonella *spp.

We need to follow rigorous standards of causal influence before claiming that a bacterium is causing an unexplained chronic disease, to avoid facing the same problem that we had with Lyme disease: a mess with open conflicts between most scientists and some atypical investigators and patient advocacy groups.

## References

[1] Maggi RG, Mozayeni BR, Pultorak EL, Hegarty BC, Bradley JM, Correa M, *Bartonella* spp. bacteremia and rheumatic symptoms in patients from Lyme disease–endemic region. Emerg Infect Dis. 2012;18:783–91. 10.3201/eid1805.11136622516098PMC3358077

[2] Raoult D, Edlinger E. The rickettsial origin of multiple sclerosis: the end of a myth [in French]. Presse Med. 1987;16:684.2953002

[3] Breitschwerdt EB, Sontakke S, Cannedy A, Hancock SI, Bradley JM. Infection with *Bartonella weissii* and detection of *Nanobacterium* antigens in a North Carolina beef herd. J Clin Microbiol. 2001;39:879–82. 10.1128/JCM.39.3.879-882.200111230398PMC87844

[4] Seriburi V, Ndukwe N, Chang Z, Cox ME, Wormser GP. High frequency of false positive IgM immunoblots for *Borrelia burgdorferi* in clinical practice. Clin Microbiol Infect. 2012;18:105–204.2236918510.1111/j.1469-0691.2011.03749.x

[5] Raoult D. Diagnostic biologique de la maladie de Lyme. Interet du Western blot. Med Mal Infect. 1990;20:163–4. 10.1016/S0399-077X(89)80277-X

# Reply: *Bartonella* spp. Bacteremia and Rheumatic Symptoms in Patients from Lyme Disease–endemic Region

**Authors:** Ricardo G. Maggi, B. Robert Mozayeni, Elizabeth L. Pultorak, Barbara C. Hegarty, Julie M. Bradley, Maria Correa, Edward B. Breitschwerdt

**Affiliations:** Author affiliations: College of Veterinary Medicine, North Carolina State University, Raleigh, North Carolina, USA (R.G. Maggi, E.L. Pultorak, B.C. Hegarty, J.M. Bradley, M. Correa, E.B. Breitschwerdt);; Translational Medicine Group, P.C., North Bethesda, Maryland, USA (B.R. Mozayeni)

**In Response:** We offer the following comments to Beard et al. (*1*) and Raoult (*2*) regarding their respective responses to our recent article (*3*). Before 1990, *Bartonella* species were not known to infect animals or humans in North America. If not for the AIDS epidemic, the expansion of literature about *Bartonella* spp. might not have occurred (Figure). In 2010, in collaboration with Raoult (*4*), we posed a question in Emerging Infectious Diseases, “Could ticks transmit *Bartonella* spp.?” That article elicited an editorial response emphasizing the lack of evidence supporting tick transmission of *Bartonella* spp. (*5*). Subsequently, *Bartonella* *birtlesii* transmission by *Ixodes ricinus* ticks was proven experimentally (*6*).

We now hope that this article will stimulate others to investigate a potential role for *Bartonella* spp. in rheumatologic diseases. Whether caused by politics or priorities, over the past 22 years, National Institutes of Health funding for *Bartonella* spp. research has been minimal and the US Centers for Disease Control and Prevention (CDC) has not critically investigated the medical impact of this genus of bacteria in US citizens. On 2 occasions, researchers at CDC declined to examine serum from these patients for antibodies against *Borrelia burgdorferi*. Because our research was not funded by any governmental agency, testing beyond our focus was not financially feasible.

We do not agree with the assertion that our study “contains serious flaws in content and underlying message, including a poorly defined study population, lack of appropriate controls, improper use of the term bacteremia, and incongruent laboratory findings.” As indicated in the Materials and Methods section of our article, a physician, B. Robert Mozayeni, recipient of a Yale residency and rheumatology fellowship and predoctoral and postdoctoral molecular immunology fellowships at the National Institutes of Health, selected all study participants. In this exploratory cross-sectional study, entry criteria were not rigid and controls were not selected at patient recruitment but were defined later from the study population. Strikingly, serologic and molecular prevalence was higher among selected patients than among occupationally high-risk veterinary professionals (*7*) tested in the same laboratory by using the same diagnostic techniques. In our article, associations were reported, causation was not argued, and caution in results interpretation was addressed in the discussion.

Bacteremia is defined as the presence of bacteria in the blood. To suggest that agar plate isolation is the only way to document bacteremia is inappropriate. *B. burgdoferi* does not grow on an agar plate, and its isolation was challenging before development of insect-based liquid growth media. PCR testing is routinely used in human and veterinary medicine to diagnose bacteremic infections by *Anaplasma, Ehrlichia,* hemotrophic *Mycoplasma*, and *Rickettsia* spp. For example, *Ehrlichia ewingii*, a recognized pathogen of canids and humans, has never been successfully isolated, whereas bacteremia is routinely diagnosed by using PCR.

In the spirit of collaboration, we have distributed *Bartonella* α Proteobacteria growth medium, an insect cell culture-based growth medium developed at and patented by North Carolina State University, to researchers around the world. Recipients included Michael Kosoy at CDC, who subsequently used this medium to isolate *Candidatus* Bartonella tamiae from febrile patients in Thailand (*8*). Subsequent studies have validated insect cell culture-based media for growth of *Bartonella* spp. For reasons that remain less than clear, there is incongruence between results of serologic testing and results of enrichment blood culture and PCR, which was addressed in our discussion and previous publications (*7*). In contrast to reports of the lack of antibodies in some bacteremic patients, we have reported specific serologic responses to infecting *Bartonella* spp. (*9*,*10*). The dated references provided by the correspondents relative to serologic testing do not address our bacteremic study population or their diseases.

We agree with Raoult that sensitive and specific diagnostic tests are critically needed to define the pathophysiology of bartonellosis. We also agree that bartonellosis is not borreliosis, and the 2 diseases should not be confused by patients, advocacy groups, Lyme disease researchers, or governmental agencies.

**Figure:** Annual worldwide number of published articles about *Bartonella* spp., 1990–2011. Data source: www.pubmed.gov.

## References

[1] Beard CB, Nelson CA, Mead PS, Petersen LR. *Bartonella* spp. bacteremia and rheumatic symptoms in patients from Lyme disease–endemic region [letter]. Emerg Infect Dis. 2012;18:1918–9.2309262610.3201/eid1811.120675PMC3559143

[2] Raoult D. *Bartonella* spp. bacteremia and rheumatic symptoms in patients from Lyme disease–endemic region [letter]. Emerg Infect Dis. 2012;18:1919.2325195210.3201/eid1811.120745

[3] Maggi RG, Mozayeni BR, Pultorak EL, Hegarty BC, Bradley JM, Correa M, *Bartonella* spp. bacteremia and rheumatic symptoms in patients from Lyme disease–endemic region. Emerg Infect Dis. 2012;18:783–91. 10.3201/eid1805.11136622516098PMC3358077

[4] Angelakis E, Billeter SA, Breitschwerdt EB, Chomel BB, Raoult D. Potential for tick-borne bartonelloses. Emerg Infect Dis. 2010;16:385–91. 10.3201/eid1603.09168520202411PMC3322042

[5] Telford SR III, Wormser GP. *Bartonella* spp. transmission by ticks not established. Emerg Infect Dis. 2010;16:379–84. 10.3201/eid1603.09044320202410PMC3322007

[6] Reis C, Cote M, Le Rhun D, Lecuelle B, Levin ML, Vayssier-Taussat M, Vector competence of the tick *Ixodes ricinus* for transmission of *Bartonella birtlesii.* PLoS Negl Trop Dis. 2011;5:e1186. Epub 2011 May 31. 10.1371/journal.pntd.000118621655306PMC3104967

[7] Maggi RG, Mascarelli PE, Pultorak EL, Hegarty BC, Bradley JM, Mozayeni BR, *Bartonella* spp. bacteremia in high-risk immunocompetent patients. Diagn Microbiol Infect Dis. 2011;71:430–7. 10.1016/j.diagmicrobio.2011.09.00121996096

[8] Kosoy M, Morway C, Sheff KW, Bai Y, Colborn J, Chalcraft L, *Bartonella tamiae* sp. nov., a newly recognized pathogen isolated from three human patients from Thailand. J Clin Microbiol. 2008;46:772–5. Epub 2007 Dec 12. 10.1128/JCM.02120-0718077632PMC2238074

[9] Oliveira AM, Maggi RG, Woods CW, Breitschwerdt EB. Suspected needle stick transmission of *Bartonella vinsonii* subspecies *berkhoffii* to a veterinarian. J Vet Intern Med. 2010;24:1229–32. 10.1111/j.1939-1676.2010.0563.x20695992

[10] Breitschwerdt EB, Mascarelli PE, Schweickert LA, Maggi RG, Hegarty BC, Bradley JM, Hallucinations, sensory neuropathy, and peripheral visual deficits in a young woman infected with *Bartonella koehlerae.* J Clin Microbiol. 2011;49:3415–7. Epub 2011 Jul 6. 10.1128/JCM.00833-1121734026PMC3165616
